# Quantifying Neurodegenerative Progression With DeepSymNet, an End-to-End Data-Driven Approach

**DOI:** 10.3389/fnins.2019.01053

**Published:** 2019-10-04

**Authors:** Danilo Pena, Arko Barman, Jessika Suescun, Xiaoqian Jiang, Mya C. Schiess, Luca Giancardo, the Alzheimer's Disease Neuroimaging Initiative

**Affiliations:** ^1^School of Biomedical Informatics, University of Texas Health Science Center at Houston (UTHealth), Houston, TX, United States; ^2^Center for Precision Health, UTHealth, Houston, TX, United States; ^3^Department of Neurology, McGovern Medical School, UTHealth, Houston, TX, United States; ^4^Center for Precision Health, UTHealth Diagnostic and Interventional Imaging, McGovern Medical School, UTHealth Institute for Stroke and Cerebrovascular Diseases, UTHealth, Houston, TX, United States

**Keywords:** Alzheimer's disease, deep learning, magnetic resonance imaging, progression, biomarkers, longitudinal, ADNI

## Abstract

Alzheimer's disease (AD) is the most common neurodegenerative disorder worldwide and is one of the leading sources of morbidity and mortality in the aging population. There is a long preclinical period followed by mild cognitive impairment (MCI). Clinical diagnosis and the rate of decline is variable. Progression monitoring remains a challenge in AD, and it is imperative to create better tools to quantify this progression. Brain magnetic resonance imaging (MRI) is commonly used for patient assessment. However, current approaches for analysis require strong a priori assumptions about regions of interest used and complex preprocessing pipelines including computationally expensive non-linear registrations and iterative surface deformations. These preprocessing steps are composed of many stacked processing layers. Any error or bias in an upstream layer will be propagated throughout the pipeline. Failures or biases in the non-linear subject registration and the subjective choice of atlases of specific regions are common in medical neuroimaging analysis and may hinder the translation of many approaches to the clinical practice. Here we propose a data-driven method based on an extension of a deep learning architecture, DeepSymNet, that identifies longitudinal changes without relying on prior brain regions of interest, an atlas, or non-linear registration steps. Our approach is trained end-to-end and learns how a patient's brain structure dynamically changes between two-time points directly from the raw voxels. We compare our approach with Freesurfer longitudinal pipelines and voxel-based methods using the Alzheimer's Disease Neuroimaging Initiative (ADNI) database. Our model can identify AD progression with comparable results to existing Freesurfer longitudinal pipelines without the need of predefined regions of interest, non-rigid registration algorithms, or iterative surface deformation at a fraction of the processing time. When compared to other voxel-based methods which share some of the same benefits, our model showed a statistically significant performance improvement. Additionally, we show that our model can differentiate between healthy subjects and patients with MCI. The model's decision was investigated using the epsilon layer-wise propagation algorithm. We found that the predictions were driven by the pallidum, putamen, and the superior temporal gyrus. Our novel longitudinal based, deep learning approach has the potential to diagnose patients earlier and enable new computational tools to monitor neurodegeneration in clinical practice.

## Introduction

Alzheimer's disease (AD) is the leading cause of dementia globally (50–75%) and is distinguished by a progressive cognitive decline (Lane et al., 2018). Currently, 5.8 million Americans suffer AD, and by 2050, this number will rise to 14 million (Alzheimer's Association, 2016). Criteria for the diagnosis of probable AD is based on subjective clinical assessments (Pfeffer et al., 1982; Marshall et al., 2015). There are multiple treatments available that can ameliorate some of the symptoms, but none of these drugs alter the course of the disease, and inevitably the dementia progresses in all patients. Neuroprotective and other disease-modifying therapies are under active development, however, to demonstrate their efficacy, sensitive, and reproducible metrics to measure disease progression are urgently needed, particularly at the earliest stage of the disease when therapies are more likely to slow the neurodegenerative progression (Aisen et al., 2017).

MRI based biomarkers for AD have been widely studied. Multiple groups have used imaging data to understand how regional brain atrophy, connectivity, or physical proximity can serve as biomarkers for dementia (Lillemark et al., 2014) or how these can be used to develop deep learning networks for AD classification (Litjens et al., 2017). These techniques are being developed to improve the accuracy of and to provide a quantitative, data-driven approach for AD disease diagnosis (Weiner et al., 2017).

In the past decade, researchers explored many avenues of the AD classification problem using machine learning. Over the years, there have been extensive reviews summarizing the state of the art of these methods (Rathore et al., 2017; Pellegrini et al., 2018). Recently, some approaches include one imaging modality (typically MRI) (Long et al., 2017), multiple imaging modalities data (Zhang et al., 2011, 2012), brain connectivity (de Vos et al., 2018), and genetic data (Peng et al., 2016). The most commonly used machine learning models are support vector machines, though there is definitely diversity in techniques (Pellegrini et al., 2018). Many of these papers focus on AD vs. CN classification, but studies are also looking at other classification tasks such as CN vs. MCI (Samper-González et al., 2018). All these methods are used for diagnosing a patient based on a model that was trained on cross-sectional data.

There is a growing interest in using machine learning to understand disease progression, and this is made possible by the available datasets for neurodegenerative disorders (Marcus et al., 2010). Researchers have used this longitudinal data to create brain development trajectories used to predict the risk of developing AD (Lawrence et al., 2017), to develop clinical symptom trajectories (Bhagwat et al., 2018) to extract essential brain features in MCI classification (Huang et al., 2017; Sun et al., 2017), and to investigate different stages of AD progression from a multi-modal imaging standpoint (Gray et al., 2012; Rodrigues et al., 2014; Nozadi et al., 2018). Similar work in Parkinson's disease used longitudinal connectome data as a marker for neurodegenerative progression (Peña-Nogales et al., 2018). In areas such as genetics, longitudinal studies have proved beneficial by revealing more single nucleotide polymorphism phenotype associations than cross-sectional studies in AD research (Xu et al., 2014). Despite the progress, existing methods use a priori hypotheses and feature engineering in their neuroimaging processing pipeline. A typical example would be the computation of the volumetric changes on a predefined number of brain areas which are used as an input to a machine learning or statistical model. This approach has two main limitations: (1), it is bound to the a-priori selection of specific brain areas, making it impractical to model disease progressions that are not fully understood; (2), any error in the estimation of the brain areas metrics would negatively influence the machine learning or statistical model (i.e., garbage in, garbage out).

To overcome the limitation of engineered features, recent studies have used the concept of feature extraction through deep learning techniques. This allows researchers to automatically extract image representations from the raw voxels specific to the outcome needed. Studies have used deep learning to tune convolutional neural networks (CNNs) on MRI images (Backstrom et al., 2018) and to process multi-modal information including genetic and neuropsychological data (Spasov et al., 2019). Further, others have used deep learning to complete other related tasks like segmentation and brain parcellation (Li et al., 2017; Gibson et al., 2018). ADNI-based machine and deep learning reviews are being written as a result (Weiner et al., 2017). However, we are not aware of end-to-end feature learning approaches to measure longitudinal changes that does not require pre-defined brain areas or region of interests for training.

In this work, we propose to use a DeepSymNet-based model, a novel end-to-end deep learning architecture, to identify longitudinal neurodegenerative progression between structural MRI images with minimal preprocessing at two-time points. We adapt the DeepSymNet architecture presented by Barman et al. (2019) to identify structural brain differences by learning time-sensitive representation on a subject-level. The imaging preprocessing pipeline required by the architecture does not use a priori brain regions or non-rigid registration algorithms making the process more robust by having fewer steps throughout the pipeline and more efficient in terms of time required to generate hypotheses and computational time than common longitudinal processing pipelines such as Freesurfer. This work has four main contributions: (1) a new neuroimaging pipeline to measure neurodegenerative progression that does not require pre-defined brain areas or region of interests for training, (2) comparable classification performance when compared with existing Freesurfer and voxel-based longitudinal pipelines for AD-relevant progression, (3) higher computational efficiency and generalizability to external dataset of mild cognitive impairment (MCI) subjects, and (4) analysis of the brain areas that drive the model's decision. This manuscript's code can be found at https://gitlab.com/lgianca/longitudinal-deepsymnet.

## Materials and Methods

Data used in the preparation of this article were obtained from the Alzheimer's Disease Neuroimaging Initiative (ADNI) database (adni.loni.usc.edu) in November 2018. The ADNI was launched in 2003 as a public-private partnership, led by Principal Investigator Michael W. Weiner, MD. The primary goal of ADNI has been to test whether serial magnetic resonance imaging (MRI), positron emission tomography (PET), other biological markers, and clinical and neuropsychological assessment can be combined to measure the progression of mild cognitive impairment (MCI) and early Alzheimer's disease (AD). The ADNI data were downloaded in November 2018 (https://ida.loni.usc.edu/login.jsp). The data were then processed according to the Brain Imaging Data Structure (BIDS) format (Gorgolewski et al., 2016). This structure allows researchers to organize their neuroimaging-related data in a concise way that allows groups to access a growing number of computational tools and pipelines compatible with the BIDS format.

In this study, we included AD, MCI, and CN subjects from ADNI who had at least two T1-weighted brain images at least 6 months apart from ADNI1, ADNIGO, and ADNI2, and this resulted in 971 patients. When patients who had more than two imaging time points, we chose the first and last sessions. The two sessions are referred to as “session 1” and “session 2” throughout the paper. Further, all the patients chosen stayed within their disease phenotype at and between the two imaging sessions and successfully passed the image preprocessing pipeline. As shown in Table 1, after the above criteria were applied, we had a total of 632 subjects between the three groups. A Kruskal-Wallis test was performed on the cohort demographics. The statistical test succeeded to reject the null hypothesis that the samples originate from the same distribution for the sex and time between sessions variables. In our comparative analysis described later, we corrected for these potential confounders.

**Table 1:** Demographics and time between imaging sessions of the AD, CN, and MCI patients used in this study (one standard deviation–s.d.).

	**AD**	**CN**	**MCI**	***p*-value**
Number of patients	212	270	150	
First session age, years [mean (s.d.)]	74.8 (7.7)	74.7 (5.9)	75.3 (7.3)	0.381
Time between sessions, years [mean (s.d.)]	1.6 (0.6)	4.4 (2.6)	3.4 (2.8)	<0.001
Sex [male, n (%)]	108 (50.9%)	138 (51.1%)	99 (66%)	<0.01
Race/Ethnicity [n (%)] -	0 (0%)	0 (0%)	0 (0%)	0.403
American Indian/Alaskan Native,	4 (1.9%)	6 (2.2%)	3 (2.0%)	
Asian	0 (0%)	0 (0%)	0 (0%)	
Native Hawaiian/Other Pacific Islander	12 (5.6%)	20 (7.4%)	8 (5.3%)	
Black/African American	193 (91.1%)	242 (89.6%)	139 (92.7%)	
White, More than one race	3 (1.4%)	2 (0.8%)	0 (0%)	
Unknown				

We will divide the methods and techniques we used into four main topics: (1) overviews of the Freesurfer-based, voxel-based, and DeepSymNet pipelines, (2) experimental design, (3) computational time analysis, (4) progression generalizability tests, and (5) confounding variable adjustment.

In the three sections, we provide an overview of the Freesurfer longitudinal pipeline, a description of the feature sets used for machine learning, and their accompanying experiments. Second, we describe the DeepSymNet-based pipeline, the experiments conducted, and the relevant brain area analysis. Third, we discuss the voxel-based machine learning method. Next, we describe comparison experiments for the above pipelines where we evaluated the computational time requirements. We then apply the models on an external MCI cohort to test the generalizability of the different pipelines and to identify progression patterns on this cohort at risk of developing AD. Finally, we describe how we evaluate the effect of potential confounders in the models.

### Data Preprocessing and Feature Set Creation Using Freesurfer Pipelines

**Figure 1:**
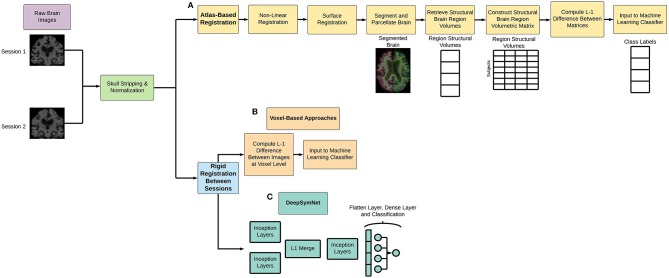
Overview of **(A)** atlas-based and non-linear registration imaging preprocessing pipeline, **(B)** voxel-based methods, and **(C)** DeepSymNet-based pipeline implementation. Note that all processes involve rigid registration to align the patient's brains longitudinally.

As seen in Figure 1 below, Freesurfer extracts the brain region volumes using multiple predefined (and time intensive) steps such as within-subject template creation, atlas registration, non-linear transformations, surface inflating. Here, we aimed to compare the model performance using two different Freesurfer version's atlas-based, longitudinal pipelines. The pipeline features will be referred to as the first feature set (FS 1) and the second feature set (FS 2), respectively. For FS 1, the “University of California San Francisco's Longitudinal Freesurfer (5.1) All Available Base Image [ADNIGO, 2]” file downloaded from the ADNI website. This independent and external source was critical to ensure that our results were comparable with already published research. The FS 2 set was taken from the output of the Freesurfer (6.0) longitudinal pipeline. A full description of the Freesurfer longitudinal pipeline is beyond the scope of this paper, and we would like to refer readers to the original publications for more information (Reuter et al., 2010, 2012; Iglesias et al., 2016). These steps are typically what researchers use in neuroimaging pipelines.

The patients between the two datasets (FS 1 and FS 2) were matched using the patient's RID (specific to ADNI's protocol) and by the closest visit date. A total of 93 subjects matched between the two datasets. For each patient, the structural MRI variables output from the pipelines were used as a feature vector. The FS 2 regional volume data came from the subcortical segmentation file (aseg.stats) and white matter parcellation files (wmparc.stats) uniquely using the region sizes. The FS 1 pipeline took data from both the header and body of the segmentation files in addition to additional statistics data from the parcellation files (e.g., thickness average and standard deviation, and surface area) which resulted in a larger feature set. For both pipelines, the same cortical and subcortical regions were investigated. Finally, the FS 2 pipeline was compared to the DeepSymNet pipeline using both the limited cohort of 93 patients and the full cohort.

### DeepSymNet-Based Pipeline Overview

#### Data Preprocessing

For the DeepSymNet preprocessing implementation, a simple longitudinal pipeline that used skull stripping, normalization, and a patient-specific alignment (Figure 1). This pipeline has several advantages for longitudinal studies. First, it uses rigid registration, which decreases computational and time costs associated with imaging pipelines. Second, there is no dependence on predefined brain regions, which enables us to take a more data-driven approach to understand progression. Third, the pipeline finds the best space to register the individual patient to which reduces the overall noise and bias that occurs when comparing samples between each other. The output of the MRI images was 182 × 218 × 182 with a resolution of 1 × 1 × 1 mm^3^. Empty voxels outside of the brain were deleted from the image and not used in the subsequent analyses.

#### DeepSymNet Architecture Overview

**Figure 2:**
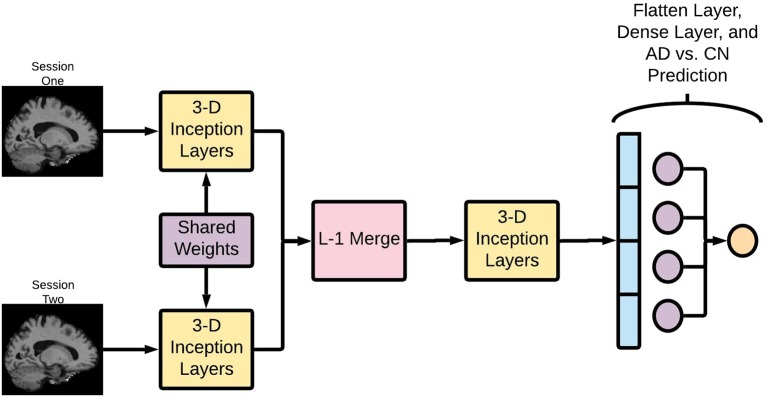
Deep Symmetry-Sensitive Convolutional Neural Network (DeepSymNet) architecture overview. Longitudinal images go through a Siamese network composed of 3-D Inception modules that share weights. These outputs are passed through a L-1 merge layer that computes differences between these two sessions. Then, this is passed through a final 3-D Inception layer to learn from these differences. Lastly, these outputs are flattened and put through a dense layer for the final AD-progression prediction.

The Deep Symmetry-Sensitive Convolutional Neural Network (DeepSymNet) architecture (Figure 2) used in this study was inspired from a model designed to identify spatial symmetries in brain angiograms (Barman et al., 2019). We applied this architecture to identify changes through time as opposed to spatial symmetries. This enables the architecture to directly learn a representation sensitive to intra-patients changes, rather than model the complex inter-patient heterogeneity and measure it over time. The model receives as input two brains at different time points, an initial step that learns a common representation between the two time points by 3-dimensional (3-D) Inception modules with weight sharing, which is then followed by a merge layer where the output of the filters is subtracted from one another, then, another set of 3-D Inception modules learn a representation sensitive to change. Finally, a max pooling and fully connected layer estimate the likelihood of a disease-relevant progression.

As AD is characterized by structural brain degeneration, a model like the one described has the potential of identifying the structural differences or progression patterns between the two MRI acquisition regardless of the brain appearance at during the first imaging session.

#### DeepSymNet Architecture Detailed

Here, we summarize the different components of DeepSymNet and walk the readers through our specific design.

##### Shared weights

Each of the two images is fed into identical neural networks before they are merged together. Additionally, these identical neural networks share weights, allowing them to learn the same patterns in the two images. This architecture allows the network to learn complex differences between the two imaging timepoints and encode information specific to that visit. Further, this part of the network can learn asymmetric patterns as neurodegeneration may differ between right and left hemispheres.

##### Inception module

The Inception modules are composed of multiresolution 3-D convolutional filters that learn to represent the T1 images at different time points according to a loss function. It should be noted that these Inception modules are the 3-D extension to what is presented by Szegedy et al. (2015), and the modules are not the same full Inception network architecture. The Inception module concatenates several parallel convolutional layers. It can be thought of as a mini network within a larger network. Since MRI images are 3-D images, 3-D convolutional filters are used within the Inception modules. For this application, the Inception module consisted of 1 × 1 × 1, 3 × 3 × 3, and 5 × 5 × 5 convolutions, followed by concatenation and a max pooling layer. These different convolution sizes enable the network to learn features at different scales, which could allow for more complex pattern recognition. Sixty-four filters have been used in the Inception modules and the network uses rectified linear unit (ReLU) activations.

##### Merge layer

An L-1 difference was then used to combine the learned convolutional features from both sessions. This encodes critical information about the structural brain changes between the timepoints, and this information is passed through another Inception layer.

##### Fully connected layers

After the merge and Inception layers, the difference between the two images is transformed into a feature vector. This vector is then passed through a fully connected layer, which creates a linear combination of the filter outputs from the penultimate layer of the network. A SoftMax operation is used as the activation function in the last layer for AD-progression prediction.

The DeepSymNet network was not designed to be fully translational invariant like the classical Inception Network (Szegedy et al., 2015). Rather, the algorithm is designed to be insensitive to the inevitable small registration inaccuracies between the two timepoints that may be present due to the rigid registration step. This is achieved through the aforementioned max pooling layers. Overall, this network is relatively shallow compared to some of the deep networks commonly reported in literature. The multiresolution filters within the inception modules in addition to the L-1 merge layer allow for complex image representation through space and time.

#### Implementation

The experiments were completed with Python (3.6.8). The DeepSymNet was implemented with Keras (2.2.4) with TensorFlow (1.12.0) as the backend. From a hardware perspective, we used Nvidia's Tesla V100 graphics cards with 32 GB RAM. The training times for each epoch varied depending on the number of model parameters, and these training lengths could range from 9 to 400 s. Each fold had 150 epochs, and a batch size of four was used. Early stopping based on a lack of improvement in validation loss for 30 epochs was also employed to reduce unnecessary computational cost. Binary cross-entropy was used as the loss function, and the Adam optimizer was used (Kingma and Ba, 2015).

#### Brain Region Relevance Analysis

In order to understand which voxels contributed to the model's predictions, the epsilon layer-wise relevance propagation (ϵ-LRP) method was used to analyze the contributions individual voxels on the final prediction (Bach et al., 2015). In summary, the ϵ-LRP method decomposes the output of deep learning architectures, on a sample level, into relevance scores in a backward fashion layer-by-layer. These scores can be projected on the pixel or voxel level of the input, which in our case is the full brain MRI, and we used a heatmap to visualize the relative magnitude of the scores. The ϵ-LRP implementation of the open-source package DeepExplain (https://github.com/marcoancona/DeepExplain) was used (Ancona and Gross, 2017).

To create this visualization that demonstrated which parts of the brain were important for DeepSymNet's predictions on AD and CN subjects, we started with the chosen model after the hyperparameter tuning test discussed in the next section. We computed the subject-level relevance with the ϵ-LRP method for both imaging sessions, and we ensured that the voxel magnitudes used were from the test set subjects. For each *n*-th patient, we added the absolute value of all the patients' voxel magnitude, *m*, for both sessions together into a single brain volume, *M*_*N*_.

$$\begin{array}{l} M_{N}=\overset{N}{\underset{i=n}{\sum }}|m_{n}| \end{array}$$

Where *m* is the voxel in the brain volume after registering to a common space and *N* represents the total number of patients.

Next, we wanted to determine the relative importance of each brain region using the magnitude of a voxel's relevance. We used the Harvard-Oxford cortical and subcortical maps to segment the brain into interpretable regions (Caviness et al., 1996). We used these respective maps to segment the voxels into different brain regions, *R*. We took the sum of all the voxel's magnitudes $m_{v}^{r}$, within each region, *r*, and then normalized this summation by the volume, *V*_*r*_, of the respective regions. This would result in the normalized volume magnitude, *M*_*r*_.

$$\begin{array}{l} M_{r}=\frac{\sum_{v=1}^{V_{r}}m_{v}^{r}}{V_{r}}\\ \;\forall r=1,2,\ldots R\\ \;\forall v=1,2,\ldots V \end{array}$$

This allowed us to see and interpret the relative progression-related importance of these different regions for the model's decision.

### Voxel-Based Machine Learning

In addition to the models discussed above, we experimented with a set of general-purpose machine learning models receiving the same input as the DeepSymNet architecture (Figure 1B). To construct the feature vector for these models, the L-1 difference between the two images was calculated, and the resulting 3-D array was flattened into one dimension and given as input to the model. Linear support vector machine and random forest classifiers were chosen as preliminary models. However, in order to avoid any model bias, we also included a strategy that incorporated robust ensemble model construction through meta-learning and Bayesian optimization (Feurer et al., 2015). This strategy was completed using the AutoML library (https://automl.github.io/auto-sklearn/master/). The architecture here is essentially optimized over the parameter space to find the “optimal” solution in order to mitigate human negligence, which offers a good baseline for our comparison.

### Experimental Design for Machine Learning Models

For the FS 1 and FS 2 sets, longitudinal based classification tasks were performed to assess the reliability between the two different pipelines. These tests used an L1-regularized logistic regression as the classifier, and the features (e.g., regional brain volumes) were scaled with respect to their inter-quartile range

$$\begin{array}{l} \frac{x_{r}-\;Q_{1}\left(x_{r}\right)}{Q_{3}\left(x_{r}\right)-\;Q_{1}\left(x_{r}\right)},\forall x_{r},\;r_{FS}=1,\;2\ldots R_{FS} \end{array}$$

where the values, x_*r*_, of brain region, *r*_*FS*_, are transformed using the quartiles *Q*.

A 10-fold cross-validation was used where the split between training and test sets were 90% and 10%, respectively. For the longitudinal classification, each patient had two feature vectors (first and last sessions), and the L-1 difference between these vectors was used as the final feature vector. The cross-validation process with random splits was conducted 100 times, and the average classification probability was taken as the average of all of these test set trials. This previously validated method offers more reliable performance on relatively small datasets as it increases the number of cross-validations without decreasing the size of the test set (Pedregosa et al., 2011; Varoquaux et al., 2017). Sensitivity and specificity metrics were calculated by choosing a cutoff point that was the minimum distance from the upper left corner in the Area Under the Receiver Operating Curve (AUC ROC). Finally, to ensure the validity of this process, tests were also conducted solely using either session 1 or session 2 data. The results from these tests were comparable to existing literature that used machine learning approaches to distinguish AD from CN with structural brain regional volumes and the ADNI dataset. Discussion of these results are beyond the scope of this paper.

For the DeepSymNet pipeline, a single 10-fold cross-validation was used due to computational constraints. Training a cross-validation approach with random splits was not feasible. Each fold contained a train and validation set, and once the fold was completed, the metrics were evaluated on a separate test set. The train, validation, and test set's proportions were 80% and 10%, and 10%, respectively. Learning rate and regularization optimization tests were conducted manually. The voxel-based approaches followed the same 10-fold cross validation and data split schematic as the DeepSymNet. Within each fold, parameters were tuned using the validation set, and the probabilities from the test sets were taken into account.

The metrics from the models trained on FS 1, FS 2, and the voxel-based methods served as the baseline metrics to compare against the DeepSymNet architecture. We chose these Freesurfer-based models for two reasons: (1) Freesurfer is arguably the most used and tested image preprocessing approach to create a representation from T1-weighted volumes. Additionally, Freesurfer has a tested and well-recognized longitudinal pipeline used by multiple research groups around the globe. (2) To ensure that we were not biased to a specific FS version's representation, we employed two sets of features.

The metrics used to evaluate the models are the AUC ROC score, balanced accuracy, sensitivity, and specificity. Further testing required the ROC curves to be compared, and these were tested for significance using the DeLong's test (DeLong et al., 1988). The ROC curves' confidence intervals were calculated using the model's predictions with a Monte Carlo resampling method with 1,000 iterations, with 80% of the data per iteration.

### Computational Time Analysis

Ultimately, we are interested in developing the underlying algorithm enabling a measuring tool for clinicians or researchers who wants to quantify an AD-relevant progression from T1-weighted brain images. Therefore, we tested the time needed to go from the raw brain images to a prediction in order to assess the feasibility of using a similar application in a clinical setting or large clinical trials. We assumed that that all ML models were already trained, as it is standard for ML applications deployment. For these tests, we used a machine with 8 CPU cores and 1 GPU. We ran 450 iterations of each pipeline and computed the mean and standard deviation of the time needed to complete. All parallelization speed-up for the Longitudinal Freesurfer-based pipeline were enabled.

### Generalizability: Detection of “AD-like” Progression Pattern on MCI Cohort

After the experiments, we wanted to see if the DeepSymNet model could apply the learned progression pattern on an external set of high-risk patients. The final DeepSymNet model was applied to a cohort of MCI patients from the ADNI protocol. Each of the 10-folds' respective best DeepSymNet model was applied to each MCI patient progression so that each patient had 10 prediction probabilities. These probabilities were averaged together for the final probability measurement. These prediction probabilities were then used to construct AUC ROC curves of MCI vs. CN. Note that the control group probabilities were taken from the test set from the 10-fold cross-validation process explained above for the MCI vs. CN AUC ROC curve. In addition, these MCI results from the DeepSymNet were compared against the same method from the logistic regression that used atlas-based registration pipeline outlined above.

### Confounding Variable Adjustment

Finally, we adjusted the DeepSymNet's probability output for confounding variables through a logistic regression method. The confounders used were the time between the two imaging sessions, gender, and the baseline age at the first imaging session.

$$\begin{array}{l} logit\;E\left(Y\right)={\beta_{0}+\;{\beta_{1}}^{*}\;X_{1}+\;\beta_{2}}^{*}\;X_{2}+\;{\beta_{3}}^{*}\;X_{3}+\;{\beta_{4}}^{*}\;X_{4}\;where\\ \;Y=Subject\;Group\;\left(e.g.\;AD,\;CN,\;MCI\right)\;\\ \;X_{1}=Time\;between\;sessions\\ \;X_{2}=Gender\\ \;X_{3}=Age\\ \;X_{4}=DeepSymNet\;Probability \end{array}$$

The feature coefficients from the logistic regression model for these variables along with their 95% confidence interval and *p*-value were reported.

## Results

In this study, we aimed to examine: (1) various models' performance on learning AD-related progression patterns, (2) image resolution and network architecture hyperparameter tests on DeepSymNet performance, (3) the evaluation of the models on an external set of MCI patients, and (4) the influence of the selected confounding variables. The purpose of the first aim was to investigate the advantages of using an end-to-end data-driven approach to understand AD progression. The second aim allows readers to understand how the model behaved with hyperparameter tuning. The third aim validated the learned AD progression-specific pattern from DeepSymNet, and the fourth aimed ensured that these output probabilities were statistically significant.

### Longitudinal Pipeline and Model Comparison

**Figure 3:**
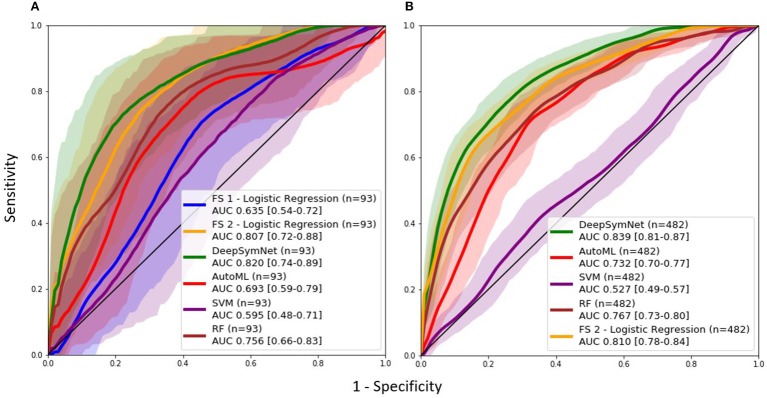
AUC ROC curves comparing performance using a **(A)** data subset allowing for a comparison with samples found in Feature Set 1 (externally computed by UCSF) and the **(B)** full dataset.

As seen in Table 2, the FS 1 and FS 2 pipelines had a differing number of structural volumetric features due to the reasons described in the Methods section. As seen in Figure 3 and Table 3 below, the DeepSymNet had the highest AUC ROC of all the methods. Of the machine learning voxel-based methods, the random forests approach performed the best. There were statistical differences in the performance between the random forest voxel-based method and the DeepSymNet. However, there was not a significant difference between the DeepSymNet and the model using Feature Set 2. This suggests that a DeepSymNet architecture learns a high-level representation of longitudinal changes that is, at least, as informative as the changes in brain regional volumes and outperforms the voxel-based general-purpose machine learning approaches tested. Finally, as seen in Table 4, the preprocessing time was much faster in the DeepSymNet and voxel-based methods vs. the traditional neuroimaging pipelines used in research.

**Table 2:** High-level comparison between the two feature sets from different imaging pipelines used to test the robustness of the new method.

	**Freesurfer version**	**Number of samples used for ML pipeline (AD/CN)**	**Number of features**
Feature set 1	5.1	93 (60/39)	340
Feature set 2	6.0	93 (60/39)	117

**Table 3:** Longitudinal models' metric evaluation for AUC ROC, sensitivity, specificity, and balanced accuracy on the full dataset.

**Model**	**AUC ROC**	**Sensitivity**	**Specificity**	**Balanced accuracy**	
FS 2 (*n* = 482)	81.0	75.9	71.9	72.3	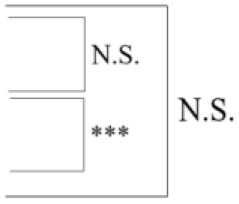
DeepSymNet (*n* = 482)	83.9	79.2	72.9	75.7	
Voxel-based random forest (*n* = 482)	76.7	68.4	72.6	64.2	

*p-values were computed from the DeLong test for correlated ROC curves to reject the null hypothesis that there is no statistical difference between the AUCs*.

N.S., not significant.

***p <0.0001.

**Table 4:** Time performance for voxel-based pipeline (including DeepSymNet) and Longitudinal Freesurfer-based pipelines to generate an AD-relevant progression metric at inference time.

**Pipeline**	**Computation time**	
	**Mean**	**Standard deviation**
DeepSymNet and other voxel-based ML Methods	6.6 min	1.2 min
Longitudinal Freesurfer-based	17.06 h	2.7 h

### DeepSymNet Hyperparameter Experiments

A non-exhaustive manual hyperparameter search for the optimal image resolution and configuration of Inception modules was conducted (Table 5). These tests were not completed with a grid-search method in order to conserve time and computational power. The resolution changes were varied between 10, 20, 25, 30, and 35 percent and were applied to the original image isotropically. Afterwards, the number of Inception modules before and after the merge layer were varied to find the optimal model.

**Table 5:** Detailed view of DeepSymNet model tuning experimental AUC ROC results.

**Experiment**	**Image resolution**	**Module configuration (Before/After)**	**AUC ROC**
Input image resolution	[10, 20, **25**, 30, 35%]	1/1	83.9
Inception module configuration	25%	[**1/1**, 1/2, 2/1, 2/2, 3/3]	83.9

*Input image resolution and Inception module configuration were examined. The tests were completed in a stepwise fashion as outlined in the table. Bolded values indicate the model chosen for further analysis*.

**Figure 4:**
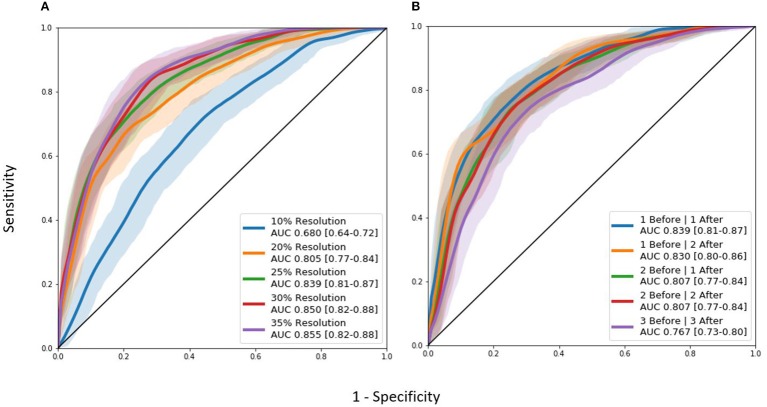
AUC ROC curves for hyperparameter tuning experiments: **(A)** Image resolution experiments. **(B)** Differing Inception module architecture experiments. “Before” and “after” labels represent the number of 3D Inception modules before and after the L-1 layer where the two timepoints are combined together.

Though the model with the 35% image resolution had a higher AUC ROC, the model that used 25% image resolution was chosen as the final model for two reasons. This model had fewer parameters (8.6 M vs. 21.2 M), and there was no statistical difference between the two curves. Several tests were conducted where the number of Inception modules before and after the merge layer was changed. As seen in Figure 4, the model that performed the best in this test contained 1 and 1 Inception modules before and after the merge layer, respectively.

### DeepSymNet Output

**Figure 5:**
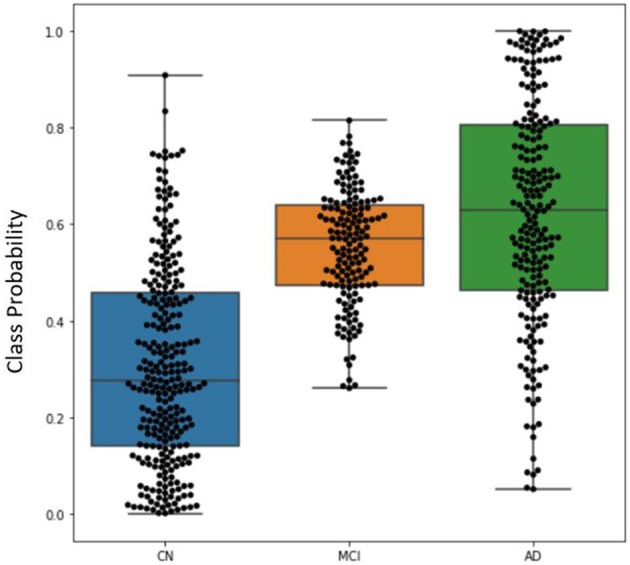
Boxplot visualizing the distribution of DeepSymNet's output probabilities for the three classes, where each dot represents the progression probability for one subject. The class probability can be used as an indicator of AD-like longitudinal progression over two timepoints.

The distribution of classification probabilities AD-like progression estimated by the DeepSymNet architecture was visualized in Figure 5 below. As expected, the MCI subjects' probabilities were in between the CN and AD groups. This indicates that the MCI group have structural progression patterns that are similar to AD. Additionally, the MCI cohort qualitatively had a smaller interquartile range compared to the other two classes.

### DeepSymNet Brain Region Relevance Analysis

**Figure 6:**
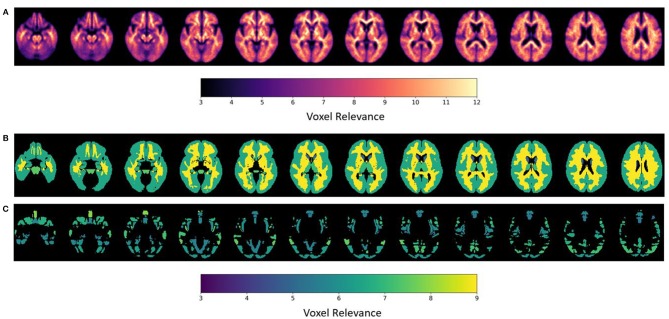
ε-LRP relevance maps indicating the contribution of each voxel and brain region to the AD-progression classification at group level. **(A)** Voxel-based relevance map; **(B,C)** normalized relevance maps mapped to brain regions; **(B)** sub-cortical **(C)** cortical. Relevance scales help denote the degree of importance for the model's decision.

**Figure 7:**
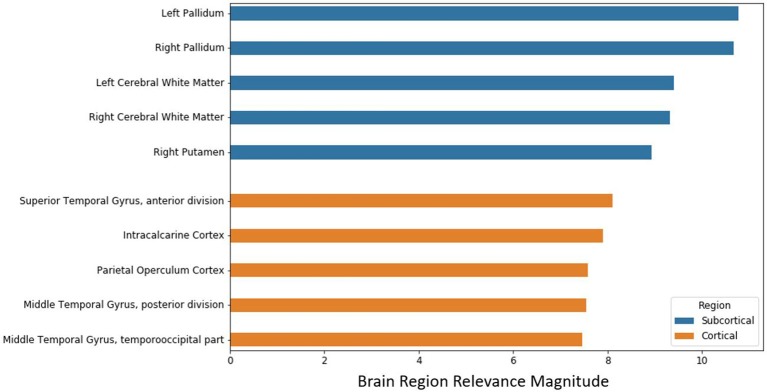
Brain regional relevance magnitudes of top five subcortical and cortical region, sorted in descending order. These values were computed by summation of all the activations within the respective region and normalization by regional volume.

After the model evaluation was completed, we wanted to understand which regions of the brain were relevant for the model's decision. The brain regions of interest are visualized both globally and based on location (subcortical vs. cortical) in Figure 6. The palladium, white matter, and putamen subcortical regions had the highest overall relevance magnitude across all the subjects and sessions. Of the cortical regions, the superior temporal gyrus cortex anterior division had the highest magnitude. The top five activated regions from the subcortical and cortical areas are summarized in Figure 7. All of the cortical and subcortical regions and their relevance magnitude can be found in the Supplementary Tables.

### Models Evaluated on an External Set of MCI Patients

**Figure 8:**
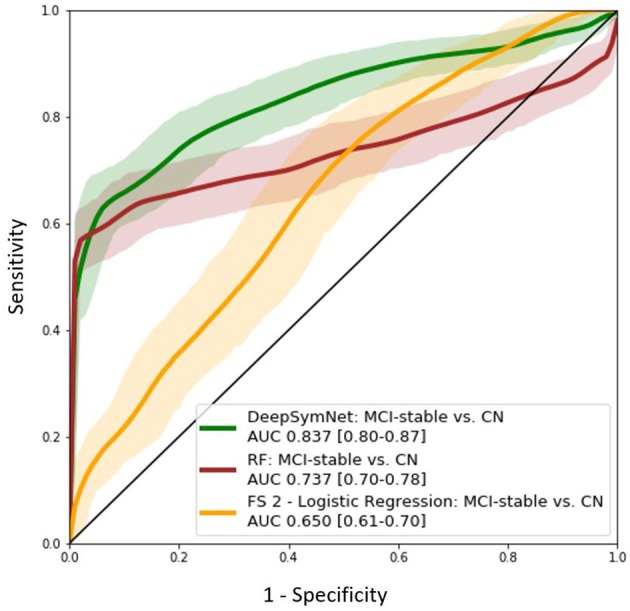
AUC ROC curves for DeepSymNet, Random forests, and logistic regression (using Feature Set 2) for the identification of an AD-like progression in the MCI (*n* = 150) and CN (*n* = 270) groups. The models were not retrained on the MCI group, as the MCI patients were used only at inference time.

Further, the final DeepSymNet model was assessed on an external set of MCI patients that DeepSymNet was never trained on. As seen in Figure 8, the AUC ROC score for identifying an AD-like progression in the MCI cohort for DeepSymNet was significantly higher than the machine learning models trained on brain regional volumes, i.e., Freesurfer-based, or voxels-based. Additionally, the AUC ROC performance is comparable to the AD vs. CN progression prediction task. This indicates DeepSymNet's ability to generalize an AD-progression specific pattern that is applicable to high-risk patients and that could not be achieved by MRI-based regional volume measures.

### Confounding Variable Adjustment

Finally, the DeepSymNet's output probabilities for the AD vs. CN and MCI vs. CN prediction tasks were adjusted using logistic regression with confounders. In both tasks, the output probabilities taken from DeepSymNet were statistically significant (*p* < 0.0001) and had the highest coefficients relative to the potential confounding variables (Table 6).

**Table 6:** Summary of the logistic regression coefficients with associated confidence interval and p-values for DeepSymNet output probability and confounding variables in both classification tasks.

**Classification task**	**Time between sessions**	**Gender**	**Baseline age**	**DeepSymNet output probability**
Progression of AD vs. CN	1.25 (0.95 to 1.54)^***^	0.03 (−0.50 to 0.56)	−0.07 (−0.08 to −0.05)^***^	4.22 (3.01 to 5.44)^***^
Progression of MCI vs. CN	−0.2 (−0.11 to 0.08)	−0.41 (−0.89 to −0.06)	−0.04 (−0.05 to −0.03)^***^	6.41 (5.01 to 7.81)^***^

*** *p <0.0001*.

## Discussion

In this study, our novel deep learning architecture, DeepSymNet, learned from temporal differences on the individual level to quantify AD progression. The DeepSymNet architecture combines the benefit of distance-based objective functions (which typically require smaller datasets) with prediction error-based objective functions (which lead to higher classification performance). The regions of the brain that drove the model's decision were visually analyzed using epsilon layer-wise relevance propagation methods. In addition, the DeepSymNet pipeline did not use typical image preprocessing steps, predefined brain regions, or non-rigid registration algorithms. These commonly used steps can be a significant source of downstream bias and computational cost. The robustness of our pipeline was benchmarked against pipelines that used atlas-based methods and baseline voxel-based machine learning models. The DeepSymNet architecture and imaging pipeline is disease-agnostic and could be used for other problems that utilize brain imaging for measuring disease progression.

AD is an ideal case study for this work as there is no current established framework to numerically quantify AD neurodegenerative progression. For clinicians to properly test new disease-modifying drugs, there is a need to develop tools that quantify AD degeneration with commonly used brain imaging scans such as MRI. Many studies have found that AD-relevant changes are visible on the T1-weighted images, and all the AD population's progression will at some point appear. This idea was supplemented with longitudinal data where our model learned from the differences between the two imaging time points.

There are several advantages to learning from time differences. First, we effectively reduce the risk of outside confounders affecting the experimental results as the individual patient's data is registered to a common space within their own specific longitudinal data. The L-1 difference used between the same subject will also reduce the magnitude of the cross-sectional patient-specific features and will instead magnify the structural differences over time. Lastly, the preprocessing method that considers individual brain morphology is in line with providing individualized precision medicine.

In order to compare the robustness of the novel longitudinal pipeline, we compared models that used either pre-defined brain region volumes or voxels as to its input against DeepSymNet. DeepSymNet could represent AD-progression comparable to region-based methods and better than voxel-based methods as indicated from the superior performance over voxel-based methods. We believe this improved performance was due to the model learning both representations of the brain and the differences between the two timepoints. Further, our deep learning model was entirely data-driven and had fewer preprocessing steps.

Two experimental hyperparameter tuning tests were conducted to improve the model performance: image resolution and Inception module architecture. Due to time and computational constraints, the authors explored through a narrow search space for tuning the model. The DeepSymNet model chosen for further analysis used images with 25% resolution and had one Inception module before and after the merge layer. This model had an AUC ROC of 0.84 (0.81–0.87).

Once tuned, DeepSymNet pipeline was then applied to an external set of MCI patients. From Figure 8, we saw an improved AUC ROC performance MCI vs. CN progression identification (0.84). This indicated that the model was able to generalize AD-specific progression patterns that are also seen in prodromal MCI patients. The DeepSymNet pipeline also achieved a performance similar to the AD vs. CN prediction task. A non-perfect classification was expected as the MCI cohort is a heterogeneous group where not all subjects will develop AD. Additionally, both classification task probabilities were statistically significant after adjusting for confounders. Finally, as seen in Figure 5, there was a clear trend where the MCI probabilities were between the CN and AD probabilities, which may indicate the degree of neurodegenerative progression.

Once the model experiments were completed, the top activated brain regions were analyzed. Previous AD studies corroborate our subcortical and cortical regional findings. Researchers found significant white matter reductions in AD patients throughout the brain, particularly in the temporal lobe (Guo et al., 2010) and elevated mean diffusivity in precuneus and entorhinal white matter microstructures (Kantarci et al., 2017). The pallidum region was found to have a significant difference in beta-amyloid burden between early and late-onset AD (Youn et al., 2017) and differences in RNA binding protein TDP-43 deposits (Josephs et al., 2016). Finally, researchers using different imaging modalities to discriminate AD patients found the pallidum and putamen to be consistently important (Rondina et al., 2018). Various frontal regions were relevant for the model decision; this might represent an advanced disease stage in the selected population.

Further, we looked at evidence surrounding our cortical region findings. An AD disease progression timeline analysis found that the superior temporal gyrus anterior division was among the top biomarkers to first become abnormal (Venkatraghavan et al., 2019). Functional connectivity analysis found decreased connectivity in the superior temporal gyrus in dementia patients including AD (Hafkemeijer et al., 2015; Schwab et al., 2018). The middle temporal gyrus has been shown to atrophy significantly in both MCI and AD patients when compared to controls in longitudinal studies (Ghazi et al., 2019) and research that combined multi-modal data types (Convit et al., 2000; Korolev et al., 2016). Finally, Guo et al. found significant gray matter volume reductions in the superior and middle temporal gyrus (2010). These subcortical and cortical structural changes might provide insight into pathophysiology process of AD and potentially serve as biomarkers for identifying those who are at risk of developing AD.

There were several limitations in this study to note. As stated in the Methods section, the first and last imaging sessions were considered for this study. Many patients that had more than two visits with MRI imaging, which indicates that there is much more data available that could be incorporated into the model. Further, the hyperparameter tuning tests were all conducted manually, in a semi-structured way, and non-exhaustively. Machine learning methods like grid search or random grid search are used to find optimal network parameters. However, due to the computational costs of these methods at training time, they were not employed. Additionally, the time between the two imaging sessions for each patient was not controlled during the sample selection, which could present itself as a confounder. The time between sessions was corrected for and controlled in the statistical analysis (Table 6). Finally, a fully external validation of these results in other AD-specific imaging datasets was not conducted.

Future studies could expand the generalizability of AD classification by using other open-source datasets such as Open Access Series of Imaging Studies (https://www.oasis-brains.org/). Also, studies could look at improving the model performance by taking a Bayesian approach for hyperparameter tuning. Other work could make use of our pipeline as a progression phenotype to assess relationship with other data sources such as cerebrospinal/blood biomarkers or genetic data. Finally, newer models such as advances in recurrent neural networks could also incorporate more time points which may provide a richer representation of a patient's progression over time.

## Conclusion

In summary, we implemented a novel pipeline based on a DeepSymNet architecture, that was able to detect an AD progression pattern by learning from structural differences of inter-subject MRI scans at two-time points. The paper's image preprocessing pipeline did not use predefined brain regions or non-rigid registration, which significantly reduced the opportunity for intermediate bias. In addition, the DeepSymNet pipeline was benchmarked against models that used standard imaging pipelines. From the brain region relevance analysis using the ε-LRP algorithm, the pallidum, putamen, and the superior temporal gyrus regions were critical in the model's final decision. Further, the model learned an AD progression pattern that was generalizable on an independent, external set of MCI patients. This architecture has the potential to be applied to multiple other applications where longitudinal changes need to be detected and measured. Finally, our pipeline can be used to improve imaging-based diagnostic systems by reducing time and computational cost.

## Data Availability Statement

The datasets generated for this study will not be made publicly available the public must request access from the Alzheimer's Disease Neuroimaging Initiative for access to the imaging data.

## Author Contributions

DP led the writing and carried out the computational experiments. AB and LG conceived the idea behind the methodology. LG and XJ reviewed and provided feedback for the methods. JS and MS assisted with the clinical relevance of the paper. All authors contributed to the writing of the final manuscript.

### Conflict of Interest

The authors declare that the research was conducted in the absence of any commercial or financial relationships that could be construed as a potential conflict of interest.
