# Supplementary material for: Quantifying Neurodegenerative Progression With DeepSymNet, an End-to-End Data-Driven Approach
Source: Front Neurosci. 2019 Oct 4;13:1053. doi: 10.3389/fnins.2019.01053 (PMC6788344; doi:10.3389/fnins.2019.01053)
Supplement: Supplementary file 1 [file Table_1.docx]

Supplementary Material

Quantifying Neurodegenerative Progression with DeepSymNet, an end-to-end data-driven approach

Danilo Pena^1^, Arko Barman^1^, Jessika Suescun^2^, Xiaoqian Jiang^1^, Mya C. Schiess^2^, Luca Giancardo^1^ and the Alzheimer's Disease Neuroimaging Initiative*

^1^School of Biomedical Informatics, University of Texas Health Science Center at Houston

^2^Department of Neurology, The University of Texas McGovern Medical School at Houston

*** Correspondence:**Luca Giancardo

[luca.giancardo@uth.tmc.edu](mailto:luca.giancardo@uth.tmc.edu)

# Supplementary Tables

**Table 1**: Full list of subcortical brain regional relevance magnitude.

| **Brain Relevance Magnitude** | **Subcortical Region** |
| --- | --- |
| 10.76535 | Left Pallidum |
| 10.67546 | Right Pallidum |
| 9.40729 | Left Cerebral White Matter |
| 9.329975 | Right Cerebral White Matter |
| 8.93802 | Right Putamen |
| 8.938007 | Left Putamen |
| 7.621991 | Left Accumbens |
| 7.473356 | Right Accumbens |
| 7.462115 | Brain-Stem |
| 7.062837 | Left Thalamus |
| 6.932809 | Left Hippocampus |
| 6.924775 | Right Thalamus |
| 6.789684 | Right Hippocampus |
| 6.581249 | Left Amygdala |
| 6.563828 | Left Cerebral Cortex |
| 6.529351 | Right Cerebral Cortex |
| 6.462989 | Right Amygdala |
| 5.144358 | Left Caudate |
| 4.709123 | Right Caudate |
| 2.782098 | Right Lateral Ventricle |
| 2.654222 | Left Lateral Ventricle |

**Table 2**: Full list of cortical brain regional relevance magnitude.

| **Brain Relevance Magnitude** | **Subcortical Region** |
| --- | --- |
| 8.108334 | Superior Temporal Gyrus, anterior division |
| 7.903124 | Intracalcarine Cortex |
| 7.579291 | Parietal Operculum Cortex |
| 7.548095 | Middle Temporal Gyrus, posterior division |
| 7.461293 | Middle Temporal Gyrus, temporooccipital part |
| 7.223988 | Lateral Occipital Cortex, inferior division |
| 7.179355 | Middle Temporal Gyrus, anterior division |
| 7.165755 | Angular Gyrus |
| 7.140806 | Inferior Frontal Gyrus, pars triangularis |
| 7.017183 | Planum Temporale |
| 6.977171 | Insular Cortex |
| 6.867739 | Cingulate Gyrus, posterior division |
| 6.866836 | Occipital Fusiform Gyrus |
| 6.847935 | Lingual Gyrus |
| 6.836203 | Occipital Pole |
| 6.8144 | Postcentral Gyrus |
| 6.783813 | Cuneal Cortex |
| 6.778783 | Supramarginal Gyrus, posterior division |
| 6.774386 | Supramarginal Gyrus, anterior division |
| 6.706776 | Precentral Gyrus |
| 6.644684 | Inferior Frontal Gyrus, pars opercularis |
| 6.545562 | Precuneous Cortex |
| 6.529464 | Central Opercular Cortex |
| 6.503387 | Cingulate Gyrus, anterior division |
| 6.461362 | Inferior Temporal Gyrus, temporooccipital part |
| 6.425344 | Temporal Occipital Fusiform Cortex |
| 6.386972 | Middle Frontal Gyrus |
| 6.295671 | Lateral Occipital Cortex, superior division |
| 6.180546 | Frontal Orbital Cortex |
| 6.170215 | Heschl's Gyrus (includes H1 and H2) |
| 6.077367 | Supracalcarine Cortex |
| 5.97827 | Temporal Fusiform Cortex, posterior division |
| 5.975193 | Frontal Pole |
| 5.903078 | Temporal Pole |
| 5.889532 | Frontal Medial Cortex |
| 5.881397 | Frontal Operculum Cortex |
| 5.816827 | Juxtapositional Lobule Cortex (formerly Supplementary Motor Cortex) |
| 5.802724 | Temporal Fusiform Cortex, anterior division |
| 5.798258 | Inferior Temporal Gyrus, posterior division |
| 5.757927 | Subcallosal Cortex |
| 5.701539 | Inferior Temporal Gyrus, anterior division |
| 5.641733 | Parahippocampal Gyrus, posterior division |
| 5.636745 | Superior Parietal Lobule |
| 5.418465 | Paracingulate Gyrus |
| 5.273092 | Planum Polare |
| 5.255703 | Parahippocampal Gyrus, anterior division |
| 5.035129 | Superior Frontal Gyrus |
